# Increasing the Safety and Storage of Pre-Packed Fresh-Cut Fruits and Vegetables by Supercritical CO_2_ Process

**DOI:** 10.3390/foods12010021

**Published:** 2022-12-21

**Authors:** Alessandro Zambon, Víctor González-Alonso, Giovanna Lomolino, Riccardo Zulli, Andreja Rajkovic, Sara Spilimbergo

**Affiliations:** 1 Department of Industrial Engineering, University of Padova, 35131 Padova, Italy; 2 Department of Agronomy, Food, Natural Resources, Animals and Environment (DAFNAE), University of Padova, 35020 Legnaro, Italy; 3 Department of Food Safety and Quality Management, University of Belgrade—Faculty of Agriculture, 11080 Belgrade, Serbia; 4 Department of Food Technology, Safety and Health, Faculty of Bioscience Engineering, Ghent University, 9000 Ghent, Belgium

**Keywords:** modified atmosphere packaging, carbon dioxide, ready-to-eat, high pressure, shelf life

## Abstract

This work presents a feasibility lab-scale study for a new preservation method to inactivate microorganisms and increase the shelf life of pre-packed fresh-cut products. Experiments were conducted on coriander leaves and fresh-cut carrots and coconut. The technology used the combination of hydrostatic pressure (<15 MPa), low temperature (≤45 °C), and CO_2_ modified atmosphere packaging (MAP). The inactivation was achieved for the naturally present microorganisms (total mesophilic bacteria, yeasts and molds, total coliforms) and inoculated *E. coli*. Yeasts and molds and coliform were under the detection limit in all the treated samples, while mesophiles were strongly reduced, but below the detection limit only in carrots. Inoculated *E. coli* strains were completely inactivated (>6.0 log CFU/g) on coconut, while a reduction >4.0 log CFU/g was achieved for carrots and coriander. For all the treated products, the texture was similar to the fresh ones, while a small alteration of color was detected. Microbiological stability was achieved for up to 14 days for both fresh-cut carrots and coconut. Overall, the results are promising for the development of a new mild and innovative food preservation technique for fresh food.

## 1. Introduction

The consumption of fresh fruits and vegetables as ready-to-eat (RTE) products has revolutionized the food industry, and it is expected to grow owing to the changing lifestyle of consumers [1]. However, when fresh-cut products are used as RTE, they usually have a limited shelf life (2–5 days), since they are affected by quick spoilage, mainly caused by oxidative enzymatic deterioration and microorganisms. Moreover, food safety in RTE food is still a challenge because a high risk of cross-contamination by pathogens can occur during the whole food chain. Good handling practices and an improvement in storage conditions and processing technologies are of primary importance to reduce the risk of outbreaks. Current technologies to increase the safety and shelf life of fresh-like products should be improved [2]. Modified atmosphere packaging (MAP) is the most widespread technology used to increase the shelf life of fresh RTE products [3]. By tuning the gas mixtures (usually N_2_, O_2,_ and CO_2_) and the specific gas barrier characteristics of the packaging materials, MAP is able to slow down the spoilage process and the microbial growth rate, thus increasing product shelf life [4]. However, MAP technology has almost no impact on the microbial inactivation [5,6]. Preservatives [7] and additional pre-treatments [8] are often used to increase the quality and safety of the products in MAP. Moreover, the possibility of combining MAP with non-thermal technologies has been investigated. For example, MAP was used in combination with gamma irradiation [9,10] and high hydrostatic pressure (HHP) [11,12] to increase the microbial inactivation and the product shelf life. However, irradiation may raise safety concerns and can be negatively perceived by consumers [13], while the very high pressure needed for HHP (<4000 bars) might induce the irreversible transformation of the texture in the fresh products [14]. 

High-pressure carbon dioxide (HPCO_2_) was extensively investigated as an alternative low-temperature pasteurization process [15], showing microbial and enzymatic inactivation in both liquid and solid products [16]. In solids, the process has been successfully applied to several food categories, such as vegetables [17,18], fruits [19,20], poultry [21], meat [22], seeds [23], and mushrooms [24]. The process is effective at supercritical conditions, meaning that it can be carried out at mild temperatures because the critical temperature of CO_2_ is relatively low (31.1 °C), thus enhancing the maintenance of heat-sensitive components [25]. In the HPCO_2_ process, liquid CO_2_ is pumped inside a high-pressure chamber where the food is placed until reaching the desired process pressure. Following this, the pressure is maintained until achieving the desired microbial inactivation. At the end of the process, the CO_2_ is released, and the treated food can be further packaged. Currently, the process itself needs post-processing packaging, which might increase the risk of cross-contamination; thus, it might represent a barrier to industrialization. 

Here, we present a preliminary study at the lab scale of a new patented method [26] for the microbial inactivation of fresh and cut RTE solid food. This new process combines the inactivation capacity of high-pressure CO_2_ within modified atmosphere packaging (HPMAP-CO_2_). The food is firstly packaged with CO_2_ as the modified atmosphere, and then the packaged sample is pressurized (pressure <15 MPa) until reaching supercritical CO_2_ (ScCO_2_) conditions using a pre-heated hydrostatic pressure vessel. The presented process is able to increase the current inactivation capacity of MAP technologies. Moreover, it avoids post-processing contamination, which might be possible after the HPCO_2_ process, because the inactivation is achieved after packaging.

The inactivation was evaluated for the naturally present microorganisms (total mesophilic bacteria, total coliforms, and yeasts and molds) and a typical contaminant and food hygiene and safety model organism (*Escherichia coli*) inoculated on the surface of the product before the treatment. The process was compared with conventional HPCO_2_ pasteurization for microbial inactivation performance on three products: coriander, carrots, and coconut. Texture and color analyses were used to assess the quality of the treated products. 

## 2. Material and Methods 

### 2.1. Sample Preparation 

Fresh carrots, coconut, and coriander were purchased from a local market in Padua, Italy, and prepared according to the literature [27,28,29,30]. All foods were kept refrigerated at 4 °C and used within a few days. Carrots were washed and cut into round pieces of 2.0 ± 0.1 g, with a thickness of 3.0–5.0 mm; coconut was washed and cut into cubes of 2.0 ± 0.1 g; and coriander leaves of similar dimensions were selected and weighed to amount to 2.0 ± 0.1 g. After that, some products were directly treated with the HPCO_2_ process, while others were packaged as explained in Section 2.2.

### 2.2. Plastic Pouches Preparation

In total, 10 cm squared pouches were crafted manually using a high gas barrier plastic film (CO_2_ permeability ≤6.5 cm^3^/m^2^/d/bar; PA/EVOH/PA/PE, EuralPack, Shoten, Belgium). Each bag was loaded with 2.0 ± 0.1 g of food products, filled with either air or CO_2_ (Rivoira, Milan, Italy), and manually closed with a thermal sealer (Impulse sealer PFS-300, Zhejiang, China). The pouch volume was 100 ± 10 mL. Gas composition inside the bags was measured with a gas analyzer (Oxybaby M+I O_2_/CO_2_, WITT, Witten, Germany).

### 2.3. High-Pressure Processes

The HPCO_2_ processes were carried out within a multi-batch high-pressure apparatus, as explained previously [31,32]. Each product was inserted in a small high-pressure vessel. After the treatment, each reactor was opened under a laminar flow hood. The processed samples were collected in sterile containers and cooled down immediately to 4 °C until further analysis. The HPMAP-CO_2_ process was carried out using the equipment previously described by Barberi et al. [33]; the packaged products were pressurized by pre-heated water. The products were packaged with 100% CO_2_ or air (HPMAP-air); HPMAP-air was used as the additional control. At the end of the process, the pouches were removed, dried gently with paper towel, and stored at 4 °C until further analysis.

Process conditions were the same for both processes: 120 bar/40 °C/20 min for carrots, 120 bar/45 °C/30 min for coconut, and 100 bar/40 °C/1 min for coriander. The process conditions were selected from the literature in order to maximize the microbial inactivation [27,28,29,30,34].

### 2.4. Microbial Count for the Natural Flora 

A standard plate count technique was used to analyze the microbial load. Samples were placed in 50 mL falcon tubes in sterility conditions, diluted 1:10 in phosphate-buffered saline solution (PBS; 0.01 M, pH 7.4; Oxoid, Hampshire, UK), and homogenized at 24.0 Hz for 1 min (Stomacher 400, International P.B.I., Milan, Italy). The solution was serially diluted (1:10) in PBS. Next, 100 µL of the appropriate dilutions was spread-plated onto the chosen media: chromogenic agar (Biolife Italiana s.r.l, Monza, Italy) for total coliforms and *E. coli*, and rose bengal (RB) (Sacco, Cadorago, Italy) for yeasts and molds; 1.0 mL was instead pour-plated into plate count agar (PCA) (Sacco, Cadorago, Italy) for the determination of the total mesophilic count. The incubation conditions were 37 °C and 24 h for *E. coli*, 30 °C and 48 h for total coliforms, 22 °C and 96 h for yeasts and molds, and 30 °C and 72 h for total mesophiles. At least three independent experiments were carried out for each single treatment condition, and the results were expressed as mean and standard deviation.

### 2.5. Culture and Inoculation of Escherichia coli

A clinically isolated *Escherichia coli* (Migula) Castellani and Chalmers (ATCC 25922) strain was used. From a frozen culture at −80 °C, the strain was resuscitated by successive overnight incubation of 100.0 µL culture in 10.0 mL of Luria-Bertani (LB) medium broth (Lennox, L3022, Sigma-Aldrich, St. Louis, MO, USA) at 37 °C. Bacterial culture was incubated at 37 °C with constant shaking (200 rpm), and the growth was carefully monitored spectrophotometrically until the stationary phase. The microbial suspension was centrifuged at 6000 rpm for 8 min; the supernatant was removed, and the pellet re-suspended in PBS reaching a final concentration of 1.0 × 10^10^ CFU/mL. Inoculated samples were spiked with 20 µL of *E. coli* suspension to reach a final inoculation level of 1.0 × 10^8^ CFU/g. Spiked samples were dried under laminar flow for 30 min before further processing. 

### 2.6. Texture Measurement

A TA.XTplus Texture analyzer (Stable Micro System, Godalming, UK) was used to measure the firmness of the carrot and coconut samples. A 500.0 N load cell, equipped with a 35.0 mm diameter compression probe, was applied; the compression rate was 2.0 mm/s, 25.0% of the initial height of the sample. For the measurement, carrots were cut into cylinders of 5.0 mm height and 10.0 mm diameter; coconut was cut into pieces and the measurement determined on the height of 10.0 mm. Results were expressed as mean value obtained from 10 different samples. The cutting test was also performed on the samples using a stainless-steel blade of 1.0 mm thickness (Lloyd Instruments LS5, Ametek, Berwyn, PA, USA) with a deformation rate of 2.0 mm/s and 75.0% strain. The cutting test was expressed as the maximum force (Newton) required to cut the sample from the beginning of the test. The parameter was acquired and processed with the Software Texture Exponent (Stable Micro Systems, Godalming, UK). 

### 2.7. pH Measurement

The pH values were measured with an electronic pH meter equipped with a Sension + 5053T penetration pH electrode (Cri-son Instruments Sa, Barcelona, Spain) for food applications, which allowed automatic temperature compensation. At least 10 determinations were executed. The pH was measured 24 h after the process.

### 2.8. Color Measurement

The color measurement was carried out with a Chroma Meter Minolta CM-600d Sensing colorimeter (Minolta Sensing Inc., Osaka, Japan), on treated and untreated samples (carrot and coconut). The device was set with a CIE (Commission Internationale de l’Éclairage), 8° standard angle observer, and D65 illuminant. The parameters lightness (L*), redness (a*), and yellowness (b*) were recorded and calculated with the CIELAB system (1978) with 400–700 nm reflectance spectrum range. The Hue angle (H*) was calculated as:

H* = tan^−1^ (b*/a*), (1)

while Chroma (C*) as:

(2) $$C*=\sqrt{{\left(a*\right)}^{2}+{\left(b*\right)}^{2}}$$

respectively. For each condition, 10 measurements were carried out.

### 2.9. Microbiological Storage Test

Samples of carrot and coconut were prepared, packaged, and treated as described in the previous sections, and then stored for 14 days at 4 °C, with monitoring of the microbial growth of the natural flora and the evolution of the gas composition of the pouches. Untreated products packaged in air (MAP-air) and CO_2_ (MAP-CO_2_) were used as the controls. Analyses were performed after the treatment (day 0) and after 3, 7, and 14 days of storage.

### 2.10. Statistical Analysis 

Statistical testing was performed in RStudio. Mean values were used to compare differences between treatments. The existence of significant differences (α = 95%) between different treatments was further considered using ANOVA and its subsequent post-hoc analysis (Tukey HSD); the Kruskal–Wallis rank-sum test and Wilcoxon rank-sum tests were used when the assumptions for an ANOVA were not fulfilled. 

## 3. Results and Discussion

### 3.1. Microbial Inactivation

**Figure 1:**
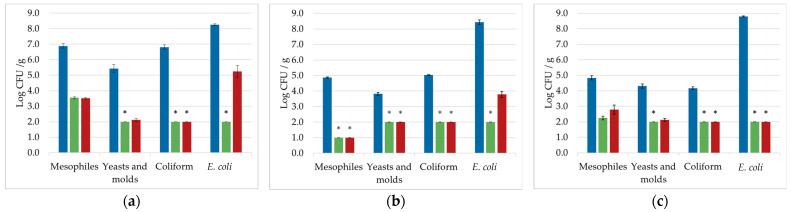
Microbial counts of total mesophiles, yeasts and molds, total coliforms, and spiked *E. coli*, measured on coriander (**a**), carrot (**b**), and coconut (**c**), in the untreated samples (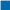), the ones treated directly with high-pressure carbon dioxide (HPCO_2_, 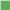) and the ones treated inside the packaging (HPMAP-CO_2_, 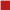). Treatment conditions were: 120 bar/40 °C/20 min for carrots, 100 bar/40 °C/1 min for coriander, and 120 bar/45 °C/30 min for coconut. * ND—Not detected. No colonies were found at the lowest dilution possible (<2.0 log CFU/g for yeasts and molds, coliforms, and *E. coli*, <1.0 log CFU/g for mesophiles).

Figure 1 shows the comparison between the microbial inactivation achieved with the two processes (HPCO_2_ and HPMAP-CO_2_) for three food products: coriander (Figure 1a), carrot (Figure 1b), and coconut (Figure 1c). 

The inactivation was demonstrated over a range of microbial groups in terms of naturally present microorganisms: total mesophiles, yeasts and molds, and total coliforms. A specific fecal indicator (*E. coli*) was also investigated by inoculation on the surface at a high concentration (1.0 × 10^8^ CFU/g). The inactivation achieved with HPMAP-CO_2_ was comparable to the one achieved with the HPCO_2_ for total mesophilic bacteria, yeasts and molds, and coliforms in all the products. Instead, a slightly higher *E. coli* inactivation was achieved for coriander and carrots with the HPCO_2_ process. For coriander, a total inactivation of the mesophiles was not possible for either treatment, while a complete inactivation was achieved for yeasts and molds with the HPCO_2_, and total coliforms using both processes. These results agree with our previous results achieved with coriander. Indeed, mesophilic bacteria were more resistant to the supercritical CO_2_ treatment compared with yeasts and molds [27]. Spiked *E. coli* on coriander was under the detection limit after the HPCO_2_ process, while almost a 3.0 log CFU/g reduction was achieved with the HPMAP-CO_2_. Bordeaux et al. showed that a complete inactivation of spiked *E. coli* was possible after the HPCO_2_ process [28]. 

For carrots, a similar inactivation for all naturally present microorganisms was achieved with the two processes. Regarding the inoculated *E. coli*, a total inactivation was achieved only after the HPCO_2_ treatment, while 4.66 log CFU/g were reduced with HPMAP-CO_2_. Tamburini et al. [35] also showed a complete inactivation when spiked carrots were treated with HPCO_2_ at 120 bar—35 °C—10 min, while Ferrentino et al. [36] showed a complete inactivation also at a lower pressure (100 bar). 

For coconut, a total inactivation of total coliforms and *E. coli* with both processes was observed, but not for the total mesophilic bacteria. Yeasts and molds were under the detection limit only after the HPCO_2_ process. In the case of mesophilic bacteria, HPCO_2_ resulted in a slightly higher inactivation compared to HPMAP-CO_2_, which nevertheless was not found to be significant. Previous studies, with HPCO_2_ at similar process conditions [30,36], showed a similar inactivation degree; however, a direct comparison might be misleading because the initial load was slightly different. Previous studies with HPCO_2_ treatment with *E. coli* on coconut showed a reduction at 60 bar/25 min/35 °C up to 5.0 log CFU/g [34]. These data indicate that *E. coli* in coconut is easily inactivated by CO_2_ even at milder conditions.

Overall results demonstrated that at a small scale the inactivation with HPMAP-CO_2_ and HPCO_2_ was comparable for the naturally present microorganisms, while the inactivation of inoculated *E. coli* was different between the two processes in carrots and coriander. The lower inactivation achieved with HPMAP-CO_2_ for *E. coli* in carrots and coriander should be further investigated with a wider number of strains and process conditions. The lower inactivation in the case of HPMAP-CO_2_ might also be explained by the presence of the packaging, which is known to influence the inactivation in the thermal pasteurization [37,38]. It might be possible that the packaging reduces the heat mass transfer, thus reducing the effective process time in which the CO_2_ is at a supercritical state. However, further studies are needed to confirm any hypothesis. 

**Figure 2:**
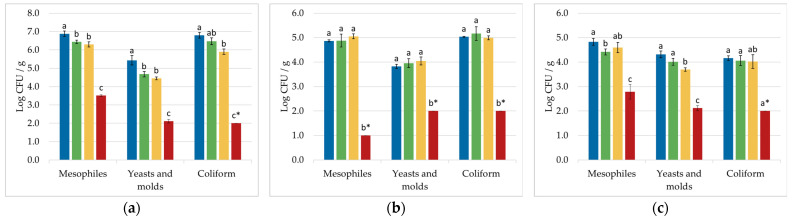
Microbial counts of total mesophiles, yeasts and molds, and total coliforms, on coriander (**a**), carrot (**b**), and coconut (**c**), in the untreated samples (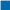), the ones treated inside the packaging with air (HPMAP-air, 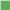), the ones packaged in CO_2_ and maintained at the processing temperature and time (TMAP-CO_2_, 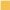), and the ones treated inside the packaging in CO_2_ (HPMAP-CO_2_, 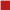). Treatment conditions were: 120 bar/40 °C/20 min for carrot, 100 bar/40 °C/1 min for coriander, and 120 bar/45 °C/30 min for coconut. Means with different small superscript letters in the same group of microorganisms are significantly different (*p* < 0.05). * ND—Not detected. No colonies were found at the lowest dilution possible (<2.0 log CFU/g for yeasts and molds, coliforms, and *E. coli*, <1.0 log CFU/g for mesophiles).

Two different controls with the HPMAP-CO_2_ process were carried out (Figure 2) to demonstrate that the microbial inactivation was due to the effect of CO_2_ at a supercritical state. In the first one, the products were packaged with air instead of CO_2,_ and then processed with HPMAP (HPMAP-air). In the second one, the products were packaged in CO_2_ and then maintained at the processing temperature for the whole processing time (TMAP-CO_2_). Microbial inactivation was not achieved when air was used instead of CO_2_ in the HPMAP process, demonstrating that the pressure itself was not responsible for any microbial reduction. The need for a simultaneous application of pressure, temperature, time, and CO_2_ was also confirmed by TMAP-CO_2_. In addition, in this case, the inactivation was negligible for all microorganisms in all the products. 

Statistical testing confirmed the significant difference (*p*-value < 0.05) for all microbial groups (mesophiles, yeasts and molds, and coliforms), in all food products tested, between the samples treated with high-pressure CO_2_ (HPCO_2_ and HPMAP-CO_2_) and the others (untreated control sample, HPMAP-air, and TMAP-CO_2_). 

The gas composition and the volume of the packages were also measured. These values were not significantly different between the treated and untreated samples. 

### 3.2. Texture, pH, and Color Analysis 

Traditional HPCO_2_ and HPMAP-CO_2_ processes were compared for texture, pH, and color change. As regards the texture, previous works suggested that fruits and vegetables treated with HPCO_2_ could lose their consistency, and thus becoming useless as an RTE product. Specifically, Valverde et al. [39] reported that pears treated with pressurized CO_2_ lost their consistency, and this loss was higher as the pressure was increased. Haas et al. [40] reported similar findings for strawberries and melons treated with HPCO_2_. 

The HPMAP-CO_2_ process induced a consistent change of color and integrity on coriander leaves, similarly to what we reported previously with HPCO_2_ [41]. A discoloration and a reduction in the firmness were also previously observed on spinach leaves after the HPCO_2_ [18] and HPP processes [42], suggesting that in general, leaves are not good candidates for high-pressure processes. On the contrary, the visual appearance of coconut and carrots was very similar to the fresh product, demonstrating that firm products could be good food products for both HPCO_2_ and HPMAP-CO_2_ processes. For this reason, only carrots and coconuts were further investigated in this study for qualitative analysis. 

The cutting force applied with a blade on a food matrix could provide indications of the fracture resistance (using the maximum force during the cutting cycle), and the rubbery behavior of the tissue, as indicated by the increase in both displacement and cutting force. A low cutting force might indicate a food matrix with a fragile structure. The results reported in Table 1 show that the untreated carrots (packaged in air (MAP-air) or CO_2_ (MAP-CO_2_)) and the ones treated with HPMAP-CO_2_ did not show significant differences for the cutting force, indicating that the process did not change the original texture and firmness of the carrots. On the contrary, samples treated with the traditional HPCO_2_ process presented a lower cutting force value, which was statistically different from the other samples. The low value after the HPCO_2_ process could be due to the de-structuring effect of the high pressures on the carrots’ tissue that could modify the cellular microstructure with consequent texture alteration [43]. In fact, as reported by Sun et al. [44], the high pressures can cause cell corruption and cell collapse up to destructive damage, cell disruption, cell wall dissolution, and overall texture degradation. These findings suggest that the HPMAP-CO_2_ process could reduce the de-structuring effect caused by the HPCO_2_ process, making the treatment more suitable for the development of minimally processed RTE products.

Coconut, on the other hand, did not show significant differences in the cutting force parameter, which was similar in all the samples (treated and untreated). This evidence may be due to the chemical composition and the particular structure of the coconut tissue which were minimally affected by the treatments applied. This finding was already demonstrated by Ferrentino et al. [19], who showed that coconut treated with HPCO_2_ maintained the same consistency compared to the fresh, untreated one. 

Finally, the textural firmness did not show significant differences for all the samples. The stress–strain curves did not present statistically significant differences in the tracings and repetitions for all the trials.

The pH for the untreated products was 6.10 and 6.17 for the carrot and coconut, respectively. These pH values are similar to the literature [19,29,45]. Neither the MAP-CO_2_ itself nor the high-pressure treatments significantly changed the pH, in contrast to what was reported in the literature for the HPCO_2_ treatment [19,29], where a slight reduction in the pH was observed after the treatment. In our case, the measurements were performed 24 h after the treatment, allowing the establishment of an equilibrium of the solubilized CO_2_ with the environment and a subsequent return to the original pH. This observation is important for the development of RTE products and consumers’ acceptance, since small changes in the pH could influence the sensorial perception. 

The color measurements are reported in Table 2 for carrots and coconut. The carrots treated with the HPMAP-CO_2_ and HPCO_2_ processes had the highest and significantly different b* values compared to the not-treated products (MAP-air and MAP-CO_2_). Significant differences were observed for the parameter a* (red/green). The highest a* values were present in the untreated products, while the processes induced lower a* values, according to what was reported by Trejo Araya et al. [46]. Based on the colorimetric data, the control samples had a more intense orange color than the treated ones. The non-treated samples did not show significant differences for the L* (lightness) parameter. On the contrary, the treated samples showed significantly lower L* values. The spatial distribution of the color was given by the hue angle (H*) and chroma (C*). The hue angle parameter showed significant differences among the carrot samples. In particular, the treated samples showed the greatest hue angle, i.e., an inclination towards yellow. Additionally, the chroma parameter had higher values for the treated samples rather than for the control. 

Furthermore, the coconut showed some significant differences for the parameters a* and L*, which were lower in the treated sample, indicating a variation in the coloring toward green. The parameters b* and C* were not affected by the treatment. On the contrary, the hue angle had significantly higher values in the coconut samples that were subjected to the treatments. 

These findings indicate that the process induced a visual change of the original color of the fresh products. For this reason, it has become more and more important to develop and improve methodologies to optimize the process minimizing the change of color, as previously reported by Barberi et al. [33]. A sensorial analysis should also be performed to confirm the perception of the consumers caused by the treatment. 

### 3.3. Microbiological Stability

A storage test at 4 °C for up to 14 days was performed for fresh-cut carrots and coconut as a proof-of-concept for the potentiality of the new HPMAP-CO_2_ technology at improving the product’s shelf life. 

**Figure 3:**
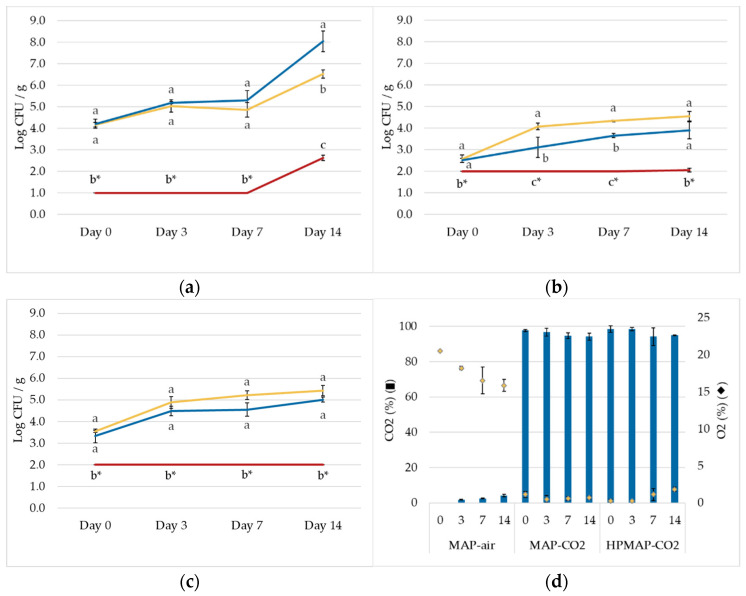
Fresh-cut carrot microbial levels of total mesophiles (**a**), yeasts and molds (**b**), and total coliforms (**c**), and gas composition (**d**), in the control packaged in air (MAP-air) (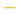), control packaged in CO_2_ (MAP-CO_2_) (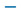), and the treated packaged in CO_2_ (HPMAP-CO_2_) (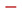), during a shelf-life trial at 4 °C. Treatment conditions were 120 bar—40 °C—20 min. Points with different small letters at the same time point represent significantly different values (*p* < 0.05). * ND—Not detected. No colonies were found at the lowest dilution possible (< 1.0 log CFU/g).

Figure 3 reports the microbial load on fresh-cut carrot for the treated HPMAP-CO_2_ and two controls (MAP-air and MAP-CO_2_). The initial microbial load was 4.2, 2.6, and 3.5 log CFU/g for mesophiles (Figure 3a), yeasts and molds (Figure 3b), and coliforms (Figure 3c), respectively. The average gas composition in terms of % CO_2_ and % O_2_ was also recorded (Figure 2d).

The results showed that the HPMAP-CO_2_ treatment was able to reduce the microbial load to undetectable levels of mesophiles, yeasts and molds, and total coliforms, and that microbial counts remained stable over a 14-day storage period. Untreated products showed microbial growth in both CO_2_ and MAP-air, eventually reaching spoilage levels. 

The gas composition of the pouches filled with 100% CO_2_ (both treated and not treated) remained stable between 95.0 and 98.0% over the storage period, while the packages filled with air underwent a decrease in O_2_ down to 16.0% by day 14, and a slight increase in CO_2_ concentration probably caused by the growth of microorganisms and the respiration of the plant tissues. 

The bacteriostatic effect of MAP-CO_2_ in the untreated product was revealed for yeasts and molds and coliforms compared with the MAP-air. Yeasts and molds have been previously reported to be sensitive to high CO_2_ concentrations during MAP [6]. On the contrary, for total mesophilic bacteria, the load of samples in MAP-CO_2_ started to be slightly higher than the MAP-air on day 7, reaching a 1.5 log higher load on day 14 compared with the MAP-air sample. This could indicate an overgrowth of lactic acid bacteria in the 100% CO_2_ samples due to the absence of oxygen [4,6]. 

Compared to traditional MAP technology, HPMAP-CO_2_ could extend the shelf life of fresh-cut carrots up to 14 days (or more). Recently, the use of pectin-coating showed the ability to increase the shelf life of fresh-cut carrots up to 12 days [47]. The technology could be potentially coupled with HPMAP-CO_2_ to obtain a synergistic effect. Microbial inactivation could be increased by tuning pressure, temperature, and treatment time, inducing a higher inactivation. Sensorial studies should be also performed to validate the consumers’ acceptance of the technology.

**Figure 4:**
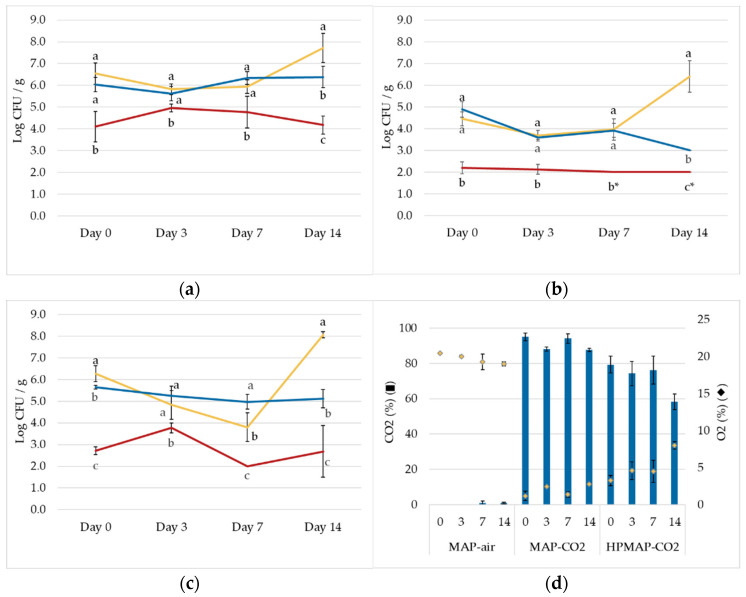
Fresh-cut coconut microbial levels of total mesophiles (**a**), yeasts and molds (**b**), and total coliforms (**c**), and gas composition (**d**), in the control packaged in air (MAP-air) (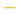), control packaged in CO_2_ (MAP-CO_2_) (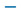), and the treated packaged in CO_2_ (HPMAP-CO_2_) (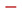), during a shelf-life trial at 4 °C. Treatment conditions were 120 bar—45 °C—30 min. Points with different small letters at the same time point represent significantly different values (*p* < 0.05). * ND – Not detected. No colonies were found at the lowest dilution possible (<1.0 log CFU/g) in at least one repetition.

Figure 4 shows the microbial counts found on fresh-cut coconut for mesophiles (Figure 4a), yeasts and molds (Figure 4b), and coliforms (Figure 4c), as well as the gas composition of the plastic pouches (Figure 4d) during a storage trial of 14 days. The initial microbial load was 6.5, 4.5, and 6.3 log CFU/g for mesophiles, yeasts and molds, and coliforms, respectively. The results showed higher variability compared to the shelf-life trial performed on carrots. On day 0, reductions of 2.2, 2.4, and 3.3 log CFU/g were achieved for mesophiles, yeasts and molds, and coliforms, respectively, compared to the untreated products, which were statistically significant (*p* < 0.05). In all three microbial groups, the counts of MAP-air at day 14 were higher than 7.0 log CFU/g for mesophiles and coliforms. However, the count up to day 7 did not show a significant increment compared to day 0, which could indicate a higher variability on the initial count due to a random contamination with the shell. However, data on day 14 demonstrated that untreated samples in MAP-CO_2_ had a lower growth compared to MAP-air, which is consistent with a bacteriostatic effect of high CO_2_ concentration MAP. The treated bags did not reach spoilage levels during the 14-day storage trial.

The HPMAP-CO_2_ coconut shelf life at 4 °C could be extended up to 14 days (or longer) in terms of microbial spoilage in comparison to samples stored in air at the same temperature. Our finding is promising for the obtainment of an extension of the shelf life of fresh-cut coconut, which in a previous study by Sinigalli et al. [48] was only 7 days.

The gas composition (Figure 4d) of the pouches loaded with fresh-cut coconut showed a different behavior compared with carrots. The concentration of O_2_ in MAP-air was reduced from 20.4% on day 0 to 19.0% on day 14. The different O_2_ consumption can be explained by the different respiration rates of coconut and carrot [49]. The CO_2_ in the MAP-CO_2_ was reduced from 95.0% CO_2_ on day 0, to 87.7% on day 14, while for the HPMAP-CO_2_, the composition was 79.0% after the treatment, and 58.0% on day 14. It is not very clear how the change in gas permeability observed compared with carrots, and additional experiments with different kinds of products and packaging materials should be performed. 

## 4. Conclusions

This work compared a new HPMAP-CO_2_ process with the traditional HPCO_2_ inactivation treatment. Despite its benefit, HPCO_2_ has never been used industrially to produce RTE foodstuff. Within this work, we proved the potential of HPMAP-CO_2_ to be used as an alternative technology to increase the safety and the shelf life of fresh RTE food. The comparison between the two technologies was performed at the lab scale on three food categories: fresh-cut carrots, fresh-cut coconut, and coriander leaves. The inactivation degree was similar between the processes, even if a slightly higher inactivation was achieved for mesophilic bacteria and inoculated *E. coli* in carrots with the HPCO_2_. The microorganisms in coconut were easily inactivated with both the processes. The microbiological shelf life was achieved up to 14 days with the HPMAP-CO_2_. HPMAP-CO_2_ was able to preserve the original texture of the fresh carrots better than HPCO_2_, while for the coconut, no differences were found after both treatments. The treatments induced a slight change in color compared to the untreated products. A further analysis should be performed to demonstrate the inactivation capacity over a wider group of spiked pathogens and the process scalability by processing bigger amounts of products. Moreover, the enzymatic activity and the retention of the nutritional properties on the treated and untreated products during the storage should be also considered in further studies. Overall, the technology results are promising for the development of a new low-temperature inactivation treatment that could be applied to strong, firm, fresh products. 

## Figures and Tables

**Table 1:** Texture analysis: effect of the different treatments on the cutting test (N) and textural firmness (J), carried out on carrots and coconut. Treatment conditions were the same as detailed in Section 3.1. Values in brackets are SD. Means with different small superscript letters in the same column are significantly different (*p* < 0.05).

Treatment	Carrot (N)	Coconut (N)	Carrot (J)	Coconut (J)
MAP-air	77.56 (12.29) ^a^	31.34 (3.93) ^a^	0.17 (0.02) ^a^	0.14 (0.02) ^a^
MAP-CO_2_	76.27 (11.48) ^a^	31.21 (3.11) ^a^	0.16 (0.01) ^a^	0.14 (0.02) ^a^
HPMAP-CO_2_	79.17 (12.39) ^a^	30.81 (4.62) ^a^	0.16 (0.03) ^a^	0.15 (0.03) ^a^
HPCO_2_	61.69 (11.72) ^b^	32.23 (6.15) ^a^	0.14 (0.02) ^a^	0.14 (0.03) ^a^

**Table 2:** Effect of the different treatments on the color of carrots and coconut, measured by CIELAB system, hue angle (H*), and chroma (C*). Treatment conditions were the same as detailed in Section 3.1. Values in brackets are SD. Means with different small superscript letters in the same column are significantly different (*p* < 0.05).

	L*	a*	b*	Chroma	Hue
Carrot					
MAP-air	62.46 (1.33) ^a^	31.70 (0.86) ^a^	42.65 (1.37) ^a^	53.15 (1.37) ^a^	53.37 (0.94) ^a^
MAP-CO_2_	60.24 (1.67) ^a^	33.59 (0.95) ^a^	45.74 (3.75) ^ab^	56.77 (3.42) ^ab^	53.62 (1.88) ^a^
HPMAP-CO_2_	60.44 (3.58) ^a^	28.31 (2.10) ^b^	49.42 (2.78) ^b^	56.98 (2.97) ^b^	60.19 (1.87) ^b^
HPCO_2_	57.99 (1.23) ^b^	26.90 (1.18) ^b^	50.33 (5.91) ^b^	57.12 (5.48) ^b^	61.68 (2.52) ^b^
Coconut					
MAP-air	83.26 (2.48) ^a^	−0.65 (0.16) ^a^	3.66 (0.60) ^a^	3.72 (0.59) ^a^	100.47 (3.54) ^a^
MAP-CO_2_	82.86 (2.34) ^a^	−0.78 (0.09) ^ac^	3.63 (0.63) ^a^	3.72 (0.61) ^a^	102.40 (2.53) ^ab^
HPMAP-CO_2_	81.70 (1.62) ^ab^	−0.87 (0.18) ^bc^	3.43 (0.89) ^a^	3.55 (0.86) ^a^	105.08 (4.07) ^b^
HPCO_2_	79.82 (2.54) ^b^	−0.98 (0.17) ^b^	4.38 (1.24) ^a^	4.49 (1.23) ^a^	103.25 (3.06) ^ab^

## Data Availability

The data are available from the corresponding author.
